# Pgltools: a genomic arithmetic tool suite for manipulation of Hi-C peak and other chromatin interaction data

**DOI:** 10.1186/s12859-017-1621-0

**Published:** 2017-04-07

**Authors:** William W. Greenwald, He Li, Erin N. Smith, Paola Benaglio, Naoki Nariai, Kelly A. Frazer

**Affiliations:** 1 Bioinformatics and Systems Biology, University of California, San Diego, 9500 Gilman Drive, La Jolla, CA 92093 USA; 2 Institute for Genomic Medicine, University of California, San Diego, 9500 Gilman Drive, La Jolla, CA 92093 USA; 3 Department of Pediatrics and Rady Children’s Hospital, University of California, San Diego, 9500 Gilman Drive, La Jolla, CA 92093 USA

**Keywords:** Hi-CChIA-PET, Chromatin conformation capture, Peak, Paired-genomic-loci, Tool suite, Bedtools, Genomic arithmetic

## Abstract

**Background:**

Genomic interaction studies use next-generation sequencing (**NGS**) to examine the interactions between two loci on the genome, with subsequent bioinformatics analyses typically including annotation, intersection, and merging of data from multiple experiments. While many file types and analysis tools exist for storing and manipulating single locus NGS data, there is currently no file standard or analysis tool suite for manipulating and storing paired-genomic-loci: the data type resulting from “genomic interaction” studies. As genomic interaction sequencing data are becoming prevalent, a standard file format and tools for working with these data conveniently and efficiently are needed.

**Results:**

This article details a file standard and novel software tool suite for working with paired-genomic-loci data. We present the **p**aired-**g**enomic-**l**oci (**PGL**) file standard for genomic-interactions data, and the accompanying analysis tool suite “pgltools”: a cross platform, pypy compatible python package available both as an easy-to-use UNIX package, and as a python module, for integration into pipelines of paired-genomic-loci analyses.

**Conclusions:**

Pgltools is a freely available, open source tool suite for manipulating paired-genomic-loci data. Source code, an in-depth manual, and a tutorial are available publicly at www.github.com/billgreenwald/pgltools, and a python module of the operations can be installed from PyPI via the PyGLtools module.

## Background

Numerous experimental methodologies have been developed in the past decade to study 3D configurations of the human genome, including Hi-C and ChIA-PET [1, 2]. These “genomic interaction” data have provided key insights into the regulation of gene expression, and suggest that chromatin interactions are driven by discrete, yet spatially-associated, epigenetic features [3, 4]. File standards and tool suites have become essential to conduct efficient bioinformatics analyses; for example, single locus information can be encoded in the BED file format and manipulated using bedtools, enabling a wide variety of bioinformatics inquiries [5]. However, it is currently challenging to fully interpret the biological impact of genomic interactions as tools do not yet exist to quickly and iteratively interrogate the extent to which both regions of paired loci are conserved across genomic datasets from diverse cell-types and contexts. While paired-genomic-loci data generated from these methodologies are widely available, the bioinformatics field has not yet developed either a file standard or analysis tools for their efficient manipulation.

There are currently several file formats for paired-genomic-loci data, however, none of these file formats were designed to enable efficient annotation and data manipulation. Existing file formats include those that encode read count information such as the matrix and the triplet sparse matrix formats [6], and others that encode the locations of paired segments and specialized metadata for particular pipelines, such as the HiFive ChromatinInteraction format [7]. Although the matrix and triplet sparse matrix formats effectively communicate coverage depth across bins of the genome, they are restricted to fixed locus bin sizes, are not human-readable, and are cumbersome for genomic arithmetic. Additionally, while the ChromatinInteraction format, and the similarly structured bedtools bedpe format [5], may appear to be suitable storage formats for integration into a genomic arithmetic pipeline, as the two loci can be written in any order within the file, programmatic manipulation is unnecessarily complicated. Finally, the triplet sparse matrix and ChromatinInteraction formats are both specialized for the specific programs for which they were designed. Thus, to facilitate genomic interaction data manipulation, allow for variable locus bin sizes within a single data set, and allow for flexible metadata important to paired-genomic-loci, a new file standard is needed.

Numerous analysis tools exist to process, normalize, or call peaks from raw reads of paired-genomic-loci data [3, 6–9], yet there is no software that performs efficient manipulation and genomic arithmetic, analogous to bedtools, for single locus data, hindering the process of annotating and comparing chromatin interactions. For example, bedtools does not provide operations for bedpe that analyze both loci simultaneously, and there are no tools for genomic arithmetic within HiFive. Furthermore, a tool for converting to the ChromatinInteraction format, or for converting from the triplet sparse matrix format to visualization formats, does not currently exist. An analysis tool suite that performs efficient manipulation and genomic arithmetic of paired-genomic-loci data would allow for more complete analyses of these datasets, and thus the potential to gain deeper biological insights about the 3D conformation of the human genome.

Here we describe a new file standard for **p**aired-**g**enomic-**l**oci data, the PGL format, and an analysis tool suite, pgltools, for genomic interaction data storage and manipulation. The PGL format supports genomic interaction data, allows for appropriate metadata, and enables efficient data manipulation. Pgltools performs genomic arithmetic on PGL files such as comparing, merging, and intersecting two sets of paired-genomic-loci, as well as integrates BED files with PGL files. Finally, we provide functions to convert other genomic interaction file formats to PGL files, and convert PGL files to multiple different visualization formats. This analysis tool suite will allow for iterative bioinformatics analyses and visualization of genomic interaction data, facilitating discovery and collaboration within the genomic interaction field.

## Implementation

Our goal was to create a file standard that can summarize the output from mapping and peak calling algorithms for chromatin interaction data derived from experiments, such as Hi-C or ChIA-PET, that is easily interpretable, shareable, and can be combined with current genomic annotation formats, such as the BED format. We first established a paired-genomic-loci file standard—the “PGL” file type— which represents each paired-genomic-loci as a single PGL entry in a human readable text file, with space in each entry for annotations, and then implemented an analysis tool suite for working with these files. Within “genomic interaction” data, the interactions between two loci (locus A and locus B) are captured—this “paired” information is preserved through the PGL file standard. PGL files require six columns in the following order: locus A chromosome, locus A start position, locus A end position, locus B chromosome, locus B start position, and locus B end position. Beyond the six columns, any user-defined annotations, such as interaction *p*-value or locus chromatin state, can be written. These annotations can be manipulated and utilized by the operations in PGLtools to gain insight into the relationship between multiple paired-genomic-loci. As annotations are unique to a file, headers can be given in files by preceding a line with “#.” Furthermore, PGL files are required to have each PGL entry written such that locus A comes before locus B based on chromosome number alphabetically (ex. chr1, chr10, chr15, chr22, chr7, chrX, chrY) and chromosome position numerically. This relationship, when combined with file sorting on each column sequentially, gives pgltools the ability to quickly merge and intersect PGL entries from PGL files. Operations for sorting PGL files, converting files to PGL files, and formatting PGL files for visualization with established programs, are also included in pgltools.

**Fig. 1:**
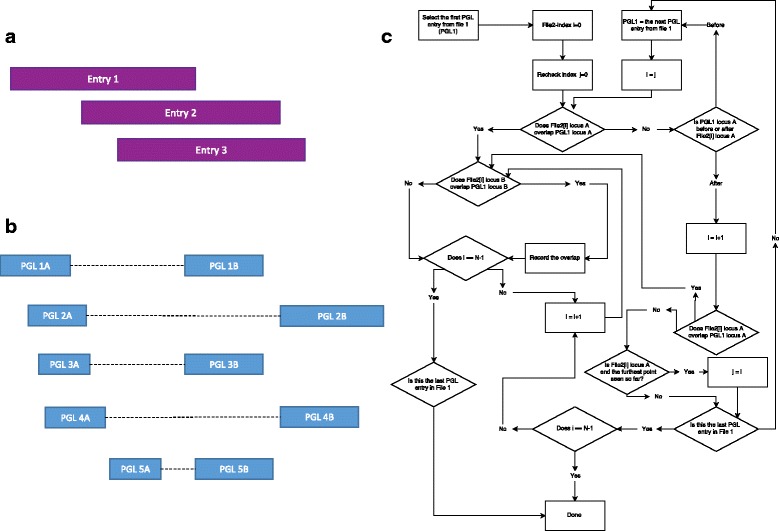
Pgltools Implementation (**a**) An example of sorted, single locus bed file entries from a file sorted by start position. As entry 1 overlaps entry 3, entry 2 must also overlap entry 3. (**b**) A pictorial representation of PGL entries in a sorted PGL file where non-sequential PGL entries overlap. Loci are shown as blocks, with *dashed lines* connecting the paired-loci comprising a single entry. Both loci A and B in PGL entries 1 and 3 overlap, and both loci in PGL entries 2 and 4 overlap. (**c**) A flowchart of the overlap function shared between many operations in pgltools. File 2 has N-1 entries. File 2 is iterated by the File2-index i. File2[i] is a PGL entry for any 0 ≤ i < N. Throughout the algorithm, PGL entries from File 2 must be checked multiple times. Therefore, to reduce the number of comparisons performed by pgltools, the Recheck Index is used to store the index at which the previous overlap iteration began. When the ends of both files are reached, the algorithm ends

Most pgltools operations utilize the same core overlap function to test for overlapping paired-genomic-loci within or between file(s). For single locus entries, such as those in sorted BED files, overlapping entries must be sequential: if entries 1 and 3 overlap, entry 2 must overlap both entries 1 and 3 (Fig. 1a). This property allows bedtools to limit of the number of features that must be compared for overlap, thus expediting analyses [5]. However, in sorted PGL files, while locus A from multiple sequential entries can overlap, locus B may not overlap (Fig. 1b). The pgltools overlap function allows for this and quickly and efficiently finds consecutive and non-consecutive entries where both locus A and locus B are overlapping. It begins by comparing the first PGLs in both files, recording if an overlap occurred in both loci, and then advances to the next PGL in File 2. These comparisons continue until the PGL from File 2 does not overlap locus A from the PGL in File 1, at which point the algorithm begins comparing the next PGL from File 1 to the first possible overlapping PGL from File 2. This repeats until the ends of both files are reached. An in-depth flow chart of the overlap operation’s control flow, as well as how the first possible overlapping PGL from File 2 is determined, is shown in Fig. 1c.

Pgltools is implemented in Python 2.7, and all operations have been tested with the pypy python compiler. As such, the UNIX package version of pgltools can be run either with CPython or pypy; the included UNIX wrapper will run pgltools through pypy if installed, or CPython if pypy is not installed. Utilizing pypy reduces memory consumption by approximately 25%, and decreases run times 5–7 fold. The pgltools suite can read from UNIX standard in, useful for stringing multiple pgltools commands together without needing to save the intermediate files, and writes to UNIX standard out, allowing it to be utilized in complex pipelines to speed up analysis of genomic interaction data. Pgltools is also available as a python module, PyGLtools, for use within pythonic pipelines, and can be installed from PyPI. As pgltools is written in Python 2.7, it is easily portable to any platform and poised for collaboration with the community.

## Results and Discussion

**Fig. 2:**
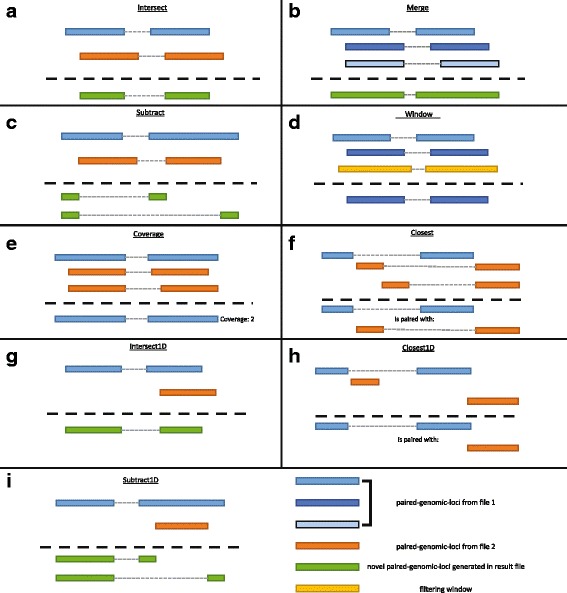
The operations of pgltools. PGL entries from file one are shown in various shades of *blue*, PGL entries from file two are shown in *orange*, and windows are shown in *yellow* (see legend at bottom right). All resulting outputs are shown below *dashed lines*, with novel entries shown in *green* and original entries shown in their original color. (**a**) The intersect operation finds overlapping paired-genomic-loci between two PGL files and returns the overlapping regions. (**b**) The merge operation combines overlapping paired-genomic-loci within a single PGL file. (**c**) The subtract operation returns the PGL entries from file one with the PGL entries from file two removed. (**d**) The window operation returns the PGL entries that fall completely within a specified genomic region. (**e**) The coverage operation returns the number of PGL entries from file two that overlap each PGL entry in file one. (**f**) The closest operation returns the closest PGL entry from file two for each PGL entry in file one. (**g**) The intersect1D operation returns PGL entries from file one that overlap regions in a bed file. (**h**) The closest1D operation returns the closest region from a bed file for each PGL entry in file one. (**i**) The subtract1D operation returns the PGL entries from file one with the regions from a bed file removed

Table 1 includes a full list of pgltools operations and their default behavior. Visualizations of these operations are provided in Fig. 2. The pgltools *intersect* operation can be used to identify either the overlap, union, or uniqueness of PGL entries between two PGL files, while preserving or combining annotations during these analyses; for example, the number of overlapping bases at each locus from each PGL entry from two PGL files can be determined. The pgltools *merge* operation can be utilized to merge overlapping PGL entries, or PGL entries within a specified distance within a single PGL file. Summary statistics, such as the number of merged entries, can be obtained through command line arguments to the *merge* operation. To determine differential PGL entries between two PGL files, the *subtract* operation has been included to remove the parts of PGL entries present in one PGL file from those present in another. Once a set of PGL entries has been determined, it is common to filter these entries to a desired genomic region—the *window* operation can be used to filter based on either or both end(s) of the PGL entries in a PGL file. To interrogate questions regarding differential coverage depth of genomic interactions, such as genetic association with interaction intensity, we provide the *samTopgl* operation, which when utilized with the *coverage* operation, will find the number of reads from a sam file that overlap each PGL entry in a PGL file (though the operation is generalizable for any two PGL files). The *closest* operation is provided for finding the closest PGL entries between two PGL files. The *expand* operation can expand both loci by a given value. In addition, as single locus genomic metadata is often analyzed together with interaction data, such as presence of a coding region, epigenetic annotation, or motif locations, we provide the *intersect1D*, *closest1D,* and *subtract1D* operations for analysis on traditional BED files and PGL files. Finally, we include helper operations both for converting files to the PGL format, including *formatbedpe* to convert a bedpe file and *formatTripSparse* to convert triple sparse matrix files, and for converting from the PGL format to packages for visualization or further analysis, such as the *conveRt* operation to convert to a file readable by the GenomicInteractions R package [10], *browser* for visualizing with the UCSC Genome Browser [11], *juiceBox* for visualizing with JuiceBox [3, 12], and *condense* and *findLoops* to create a BED file of either the discrete loci or interior regions of each PGL.

**Table 1:** Summary of operations provided in pgltools

Method	Description
intersect	Find overlapping paired-genomic-loci from two PGL files
merge	Merge nearby paired-genomic-loci within a single file and produce a column containing summary statistics requested through passed parameters (-c and -o)
subtract	Find parts of paired-genomic-loci from a PGL file that do not overlap another PGL file
window	Filter a PGL file to a particular genomic region
samTopgl	Converts a sam file to a PGL file
coverage	Find the coverage of a PGL file on another PGL file; usually used to find the coverage of reads from a PGL file derived from a sam file on a set of PGLs. The paired-genomic-loci from file 2 only need to overlap the paired-genomic-loci from file 1.
closest	Find the closest paired-genomic-loci from a PGL file for each paired-genomic-loci in another PGL file
expand	Expand both loci by a given size
intersect1D	Find the paired-genomic-loci that overlap regions from a bed file
closest1D	Find the closest paired-genomic-loci to a set of regions from a bed file
subtract1D	Find the parts of paired-genomic-loci that do not overlap regions from a bed file
sort	Sorts a PGL file for use with other PGLtools operations
formatbedpe	Convert a bedpe-like file to a PGL file
formatTripSparse	Convert a triplet sparse matrix file set to a PGL file
conveRt	Formats the PGL file for use with the GenomicInteractions R package
browser	Format a PGL file to be viewed in the UCSC Genome Browser
juicebox	Format a PGL file to be viewed in juicebox
condense	Convert a PGL file to a BED file with two entries for each PGL entry.
findLoops	Convert a PGL file to a BED file with an entry containing the region from the start of anchor A to the stop of anchor B for intra-chromosomal PGLs, and an entry for each anchor for inter-chromosomal PGLs.

By combining the operations within pgltools, one can quickly and easily interrogate biological functionality in the context of chromatin interaction data. For example, by combining the *intersect1D* and *merge* operations, it is possible to determine the different chromatin annotations for each locus of each PGL entry (which could then be further filtered to determine 3D interactions between chromatin states of interest, e.g. promoter-enhancer). Additionally, pgltools can be used to find overlaps between chromatin interactions and other types of paired data. For example, one could create a PGL file from a list of expression Quantitative Trait Loci (**eQTLs**) and their corresponding target genes (**eGenes**), and utilize the *intersect* operation to determine if any pairs of eQTL and eGenes fall within a chromatin interaction. Example pipelines for these scenarios can be found on the pgltools github.

## Conclusions

Pgltools is an open source software analysis tool suite for interacting with the PGL file standard for paired-genomic-loci. Pgltools can read from and writes to UNIX standard in and standard out, and can be run quickly in both CPython and pypy. A python module version, PyGLtools, is available for use within pythonic pipelines. The cross-platform nature of python poises pgltools for community contribution, and makes it easy to install and utilize.

## Abbreviations

eQTLs: Expression quantitative trait loci
NGS: Next-generation sequencing
PGL: Paired-genomic-loci
